# Association between tumor necrosis factor alpha rs1800629 polymorphism and risk of osteoarthritis in a Chinese population

**DOI:** 10.1590/1414-431X20187311

**Published:** 2018-05-28

**Authors:** Jie Chen, Yu Wu, Jiannong Yu, Jinming Shen

**Affiliations:** Department of Orthopaedics, Zhejiang Provincial Hospital of Traditional Chinese Medicine, Hangzhou, Zhejiang, China

**Keywords:** TNF-α, Osteoarthritis, Polymorphism, Meta-analysis

## Abstract

Osteoarthritis (OA) is the most common degenerative disease affecting articular cartilage. Some studies indicate that tumor necrosis factor alpha (TNF-α) gene rs1800629 polymorphism was associated with OA risk among Caucasian populations. To examine the role of this candidate gene in Asian populations, we conducted a hospital-based case-control study involving 257 knee OA patients and 305 controls in a Chinese population. Genotyping was performed using a custom-by-design 48-Plex single nucleotide polymorphism (SNP) Scan^™^ kit. Our study indicated that the AA genotype of TNF-α rs1800629 polymorphism was associated with increased risk of OA. Subsequently, we conducted a meta-analysis and found that rs1800629 polymorphism increased the risk of OA in the recessive and homozygous models. Stratification analysis of ethnicity also obtained a significant association among Asian populations. In conclusion, TNF-α rs1800629 polymorphism confers susceptibility to OA, especially among Asians. Larger studies with more diverse ethnic populations are needed to confirm these results.

## Introduction

Osteoarthritis (OA) is characterized by loss of joint cartilage and loss of function primarily in the knees, affecting 9.6% of men and 18% of women over 60 years of age (1). This disease could contribute to functional decline in quality of life with heavy health care and society costs (2). Risk factors can be divided into person-level factors, such as age, sex, obesity, genetics, race/ethnicity, and diet, and joint-level factors including injury, malalignment, and abnormal loading of the joints (3). Twin and family aggregation studies have demonstrated that OA and its endophenotypes are largely genetically determined, but the underlying genetic variants are mostly unknown (4–6).

Tumor necrosis factor alpha (TNF-α) is a proinflammatory cytokine, which plays an important role in the pathogenesis of OA (7). A study conducted by Chen et al. demonstrated that interleukin-1β (IL-1β) and TNF-α increased stiffness and impaired contractile function of articular chondrocytes (8). Circulating levels of interleukin-6 (IL-6) and TNF-α were associated with knee OA in older adults (9). *In vitro*, pro-inflammatory IL-1β and/or TNF-α up-regulate matrix metalloproteases-1 and -3 mRNA in chondrocyte subpopulations potentially pathogenic in OA (10). In addition, mRNA expression of TNF-α in knee OA patients was 1.56 times greater than in healthy controls. Based on these observations, TNF-α may provide insight into the etiology of OA.

TNF-α is located in the chromosome 6:31,575,567-31,578,336. The rs1800629 polymorphism in the TNF-α gene is also known as the TNF-308 single nucleotide polymorphism (SNP). Occasionally the rs1800629(A) allele is referred to as 308.2 or TNF2, with the more common G allele being 308.1 or TNF1. The A allele is associated with higher levels of TNF expression (Ensemble database). Recently, many studies assessed the association between TNF-α rs1800629 polymorphism and OA risk, but conflicting results were yielded (11–20). Some studies observed positive findings between rs1800629 polymorphism and OA risk, but others did not. Therefore, to validate whether this single nucleotide polymorphism confers susceptibility to OA in a Chinese population, we conducted this hospital-based case-control study.

## Material and Methods

### Study subjects

A total of 257 patients diagnosed with knee OA and 305 age-matched unrelated healthy controls were recruited in this study. The diagnosis of knee OA followed the criteria of the American College of Rheumatology, and radiographic findings of OA were categorized into Kellgren-Lawrence (KL) grades 1, 2, 3 or 4 (21). Patients with other diseases such as gout, rheumatoid arthritis, systemic lupus erythematosus, psoriasis, and developmental dysplasia were excluded from this study. Control subjects were selected among people without personal and family history of OA.

We obtained approval for the study protocol from the Ethics Committee of Zhejiang Provincial Hospital of Traditional Chinese Medicine (China). This study was conducted in line with the Declaration of Helsinki. All patients provided written informed consent prior to their participation.

### DNA extraction and genotyping

Two milliliters of peripheral blood from each patient was collected using vacutainers and transferred to test tubes containing ethylenediaminetetraacetic acid (EDTA). DNA was extracted from blood samples using the QIAamp DNA Blood Mini Kit (Qiagen, Germany) and stored at −20°C. SNP genotyping was performed using a custom-by-design 48-Plex SNP Scan^™^ kit (Genesky Biotechnologies Inc., China).

### Statistical analysis

Differences in demographic characteristics, variables, and genotypes of the TNF-α rs1800629 polymorphism were evaluated using a chi-squared test. The association between TNF-α rs1800629 polymorphism and risk of OA was estimated by computing odds ratios (ORs) and 95% confidence intervals (CIs) using logistic regression analyses. The Hardy-Weinberg equilibrium (HWE) principle was tested by a goodness-of-fit chi-squared test, to compare the observed and expected genotype frequencies among controls. Statistical analyses were performed using the SAS software package (version 9.1.3; SAS Institute, USA). To fully investigate the association of TNF-α rs1800629 polymorphism with OA, we also conducted a meta-analysis, which was performed using the Stata 11.0 software (StataCorp, USA).

## Results

### Characteristics of the study population

Patient demographics and risk factors in OA are summarized in Table 1. Subjects were adequately matched for age and sex (P=0.854 and P=0.653, respectively). There was no significant association in the subgroup analysis of BMI. Kellgren-Lawrence (KL) grade 2 accounted for 44.4% of participants.

**Table 1.** Patient demographics and risk factors for osteoarthritis.

Variable	Cases (n=257)	Controls (n=305)	P
Age (years)	54.35±15.34	54.59±15.40	0.854
Male/Female	64/193	71/234	0.653
Body mass index	22.92±4.05	22.34±3.95	0.087
Kellgren-Lawrence grade			
1	15 (5.8%)		
2	114 (44.4%)		
3	92 (35.8%)		
4	36 (14.0%)		

Data are reported as means±SD or number and percentage. Statistical analysis was done with the chi-squared test.

### Association between TNF-α rs1800629 polymorphism and OA risk

The genotype distributions of TNF-α rs1800629 polymorphism are presented in Table 2. Genotype distributions of the controls in this study conformed to the HWE (P=0.043). Logistic regression analyses revealed that the AA genotype of rs1800629 polymorphism was associated with significantly increased risk of OA compared to the GG genotypes (AA *vs* GG: OR=2.06, 95%CI=1.11–3.80, P=0.022; Table 2).

**Table 2.** Logistic regression analysis of associations between TNF-α rs1800629 polymorphism and risk of osteoarthritis.

Genotype	Cases (n=257)		Controls (n=305)		OR (95%CI)	P
	n	%	n	%		
GA *vs* GG	96/120	37.4/46.7	140/142	45.9/46.6	0.81 (0.57,1.16)	0.250
AA *vs* GG	33/120	12.8/46.7	19/142	6.2/46.6	2.06 (1.11,3.80)	**0.022**
GA+AA *vs* GG	129/120	50.2/46.7	159/142	52.1/46.6	0.96 (0.69,1.34)	0.812
AA *vs* GA+GG	33/216	12.8/84.0	19/282	6.2/92.5	2.27 (1.26,4.10)	**0.007**
A *vs* G	162/336	31.5/65.4	178/424	29.2/69.5	1.15 (0.89,1.48)	0.290

Genotyping was successful in 249 cases and 301 controls. Bold data are statistically significant (P<0.05).

### Meta-analysis results

The characteristics of the studies exploring the associations between TNF-α rs1800629 polymorphism and the risk of OA are listed in Table 3. Four Asian studies (including this study) and 7 Caucasian studies were identified in this meta-analysis. The Newcastle-Ottawa Scales (NOS) scores ranged from 6 to 8 stars, suggesting that studies were of high methodological quality.

**Table 3.** Characteristics of included studies for different genotypes.

Author, year	SOC	Ethnicity	Type of OA	GG		GA		AA		HWE	NOS
				case	control	case	control	case	control		
Moos, 2000 (13)	HB	Caucasian	Knee/Hip	36	166	18	74	1	0	0.005	7
Romero, 2002 (17)	HB	Caucasian	Knee	28	28	3	0	0	0	N/A	7
Sezgin, 2008 (11)	HB	Caucasian	Knee	121	72	26	12	4	0	0.481	6
Han, 2012 (14)	PB	Asian	Knee	79	258	188	33	34	0	0.305	6
Valle, 2012 (18)	PB	Caucasian	Knee	44	93	6	7	0	0	0.717	5
Ji, 2013 (19)	PB	Asian	Knee	143	253	50	50	7	2	0.782	6
Cheng, 2013 (20)	PB	Asian	Knee	143	253	50	49	7	3	0.716	7
Hamalainen, 2014 (15)	PB	Caucasian	Hand	231	578	82	173	7	13	0.989	8
Abdel, 2017 (16)	PB	Caucasian	Knee	180	115	25	82	5	13	0.749	7
This study	HB	Asian	Knee	120	142	96	140	33	19	0.043	6

SOC: source of controls; OA: osteoarthritis; PB: population-based controls; HB: hospital-based controls; NOS: Newcastle-Ottawa Scale; HWE: Hardy–Weinberg equilibrium.

**Figure 1.**
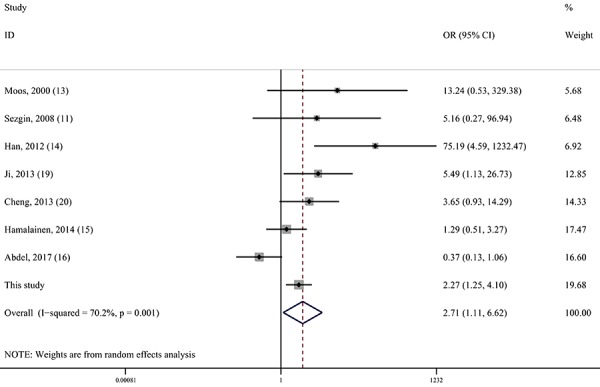
Forest plot showing odds ratio for the associations between rs1800629 polymorphism and osteoarthritis risk (AA *vs* GA+GG).

**Figure 2.**
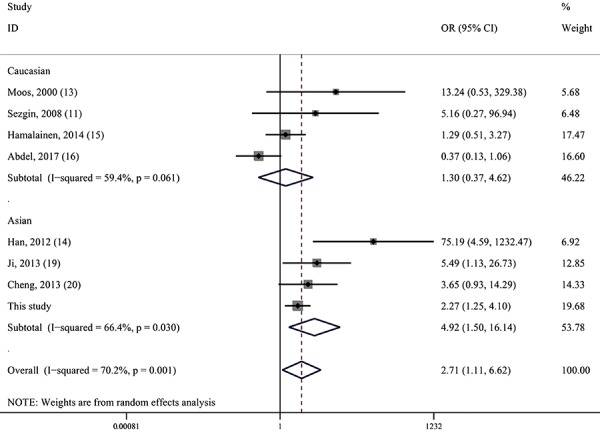
Stratification analyses of ethnicity between rs1800629 polymorphism and osteoarthritis risk (AA *vs* GA+GG).

Our meta-analysis revealed that TNF-α rs1800629 polymorphism was associated with the increased risk of OA under recessive and homozygous models (AA vs GA+GG: OR=2.71, 95%CI=1.11–6.62, P=0.029; Figure 1). In the ethnicity subgroup analysis, we found that this SNP showed correlation with increased risk among Asian populations (AA vs GA+GG: OR = 4.92, 95 %CI=1.50–16.14, P=0.009; Figure 2), but not in Caucasian populations (Table 4).

**Table 4.** Meta-analysis of association between TNF-α rs1800629 polymorphism and the risk of osteoarthritis.

Comparison	Studies	OR (95%CI)	P-value	P for heterogeneity
A *vs* G				
Caucasian	7	1.14 (0.59,2.20)	0.707	<0.001
Asian	4	2.69 (0.97,7.47)	0.058	<0.001
Total	11	1.65 (0.90,3.05)	0.107	<0.001
AA+GA *vs* GG				
Caucasian	7	1.10 (0.52,2.31)	0.802	<0.001
Asian	4	2.97 (0.80,11.11)	0.105	<0.001
Total	11	1.69 (0.81,3.54)	0.165	<0.001
AA *vs* GA+GG				
Caucasian	4	1.30 (0.37,4.62)	0.685	0.061
Asian	4	4.92 (1.50,16.14)	0.009	0.030
Total	8	2.71 (1.11,6.62)	0.029	0.001
AA *vs* GG				
Caucasian	4	1.33 (0.29,6.15)	0.718	0.014
Asian	4	7.05 (1.43,34.68)	0.016	0.001
Total	8	3.20 (1.08,9.55)	0.037	<0.001
GA *vs* GG				
Caucasian	7	1.05 (0.51,2.18)	0.886	<0.001
Asian	4	2.63 (0.70,9.90)	0.154	<0.001
Total	11	1.57 (0.76,3.25)	0.224	<0.001

## Discussion

TNF-α stimulated chondrocyte responses that promote catabolism of type II collagen and proteoglycans, thereby compromising cartilage extracellular matrix integrity and tissue homeostasis in OA (22). TNF-α could increase the expression of cytokines and chemokines (MMP-1, MMP-3, MMP-13, and ADAMTS-4) in synovial cells and renew inflammatory mediators (23,24). Some studies demonstrated that TNF-α induced chondrocyte death and hampered the migration of chondrogenic progenitor cells (25–27). In addition, studies revealed that TNF-α rs1800629 polymorphism was associated with high expression of TNF-α in knee OA (12). In this case-control study, we found that this SNP increased the risk of OA in a Chinese population. We also validated this significant association in the overall populations by a meta-analysis. Subgroup analysis of ethnicity indicated that this SNP increased the risk of OA among Asians, but not among Caucasians.

The relationship between TNF-α rs1800629 polymorphism and OA risk have been discussed previously (11–20). However, the results of these studies were contradictory. In 2000, Moos et al. (13) failed to find the association between TNF-α rs1800629 polymorphism and OA susceptibility among Caucasians. The finding was also observed in the subsequent studies of Caucasian populations (11,15,17,18). However, a recent study from Egypt obtained an association between rs1800629 polymorphism and knee OA (16). Subsequent studies (14,19,20) from Asia also found that this SNP was associated with increased risk of OA. Munoz-Valle et al. (12) found that G allele of TNF-α rs1800629 polymorphism was associated with high mRNA and soluble expression in knee OA patients. However, the TNF-α rs1800629 genotypes did not show statistical difference between cases and controls. In addition, the sample size of the study by Munoz-Valle et al. (12) was very small. Thus, we could not exclude the possibility that their findings might be false-positive. We hypothesized that rs1800629 polymorphism may be in linkage disequilibrium with other potentially functional variants in TNF-α gene or closely linked susceptibility genes, which may contribute to the contradictory findings. In this study, our data showed that AA genotype was associated with the risk of OA. We do not know whether AA genotype is associated with higher production of TNF-α expression in this study, which warrants further study to validate the results. Small sample size, low statistical power, and/or clinical heterogeneity may account for the disparities of the above studies. In order to overcome the limitations of individual studies and resolve inconsistencies, Kou and Wu (28) reviewed 7 studies with 983 cases and 1355 controls and conducted a meta-analysis to derive a more precise estimation of the effect of TNF-α rs1800629 polymorphism on OA risk in 2014. Their results revealed that this polymorphism increased the risk of OA in the allelic and recessive models (28). Ethnic subgroup analysis indicated that rs1800629 polymorphism was associated with the risk of OA among Asian populations (28). To date, there are 4 studies (3 Caucasian studies and this study) with moderate sample sizes (12,15,16).

We re-evaluated the role of TNF-α rs1800629 polymorphism in the risk of OA by a meta-analysis. Our conclusion is almost in line with the conclusion of the meta-analysis of Kou and Wu (28) who reported that A allele or AA genotype of this SNP increased the risk of OA. However, we only observed that AA genotype was associated with increased risk of OA, but not A allele. We believe our meta-analysis was more robust than the previous meta-analysis (28) due to larger sample size. Stratification analysis of ethnicity in this meta-analysis suggested that this SNP increased the risk of OA among Asians, but not among Caucasians. Several possible explanations for different findings of this SNP between Asians and Caucasians may account for these contradictory findings. First, genetic heterogeneity for OA may exist among different populations. Second, the discrepancy may be explained by clinical heterogeneity between the different ethnicities. Third, the sample sizes, different genotyping methods, and random errors may also be potential reasons for these disagreements. Further studies with larger sample sizes among other races are needed to verify these findings.

Some limitations of this study should be taken into consideration. One, confounding factors may have affected the results, such as smoking habit and weight. Two, the sample size of this study was not large, which might make our work underpowered. Three, lack of available original data prevented adjustment for other covariates such as age, gender, and lifestyle. Four, high heterogeneity was observed in this meta-analysis. Five, included studies mainly involved Asians and Caucasians and results may not be applied to other racial groups. Six, we did not evaluate whether AA genotype is associated with higher production of TNF-α expression in this study. Seven, only one SNP was investigated in this case-control study. Finally, there was no AA mutant genotype in some studies of this meta-analysis.

In summary, TNF-α rs1800629 polymorphism may be associated with increased risk of OA. The meta-analysis also revealed an association between this SNP and OA risk. More studies with larger sample sizes are warranted to confirm this relationship.
